# Shenmai Injection Protects Against Doxorubicin-Induced Cardiotoxicity *via* Maintaining Mitochondrial Homeostasis

**DOI:** 10.3389/fphar.2020.00815

**Published:** 2020-06-05

**Authors:** Lin Li, Jinghao Li, Qilong Wang, Xin Zhao, Dongli Yang, Lu Niu, Yanze Yang, Xianxian Zheng, Limin Hu, Yuhong Li

**Affiliations:** ^1^Institute of Traditional Chinese Medicine, Tianjin University of Traditional Chinese Medicine, Tianjin, China; ^2^Key Laboratory of Pharmacology of Traditional Chinese Medical Formulae, Ministry of Education, Tianjin University of Traditional Chinese Medicine, Tianjin, China; ^3^Tianjin Key Laboratory of Chinese Medicine Pharmacology, Tianjin University of Traditional Chinese Medicine, Tianjin, China

**Keywords:** doxorubicin, Shenmai injection, cardiotoxicity, PI3K/Akt signaling pathway, AMP-activated protein kinase, mitochondrial dynamics

## Abstract

Shenmai injection (SMI), as a patented traditional Chinese medicine, is extracted from Panax ginseng and Ophiopogon japonicus. It commonly used in the treatment of cardiovascular disease and in the control of cardiac toxicity induced by doxorubicin (DOX) treatment. However, its anti-cardiotoxicity mechanism remains unknown. The purpose of this study was to investigate the underlying mitochondrial protective mechanisms of SMI on DOX-induced myocardial injury. The cardioprotective effect of SMI against DOX-induced myocardial damage was evaluated in C57BL/6 mice and H9c2 cardiomyocytes. *In vivo*, myocardial injury, apoptosis and phosphoinositide 3-kinase (PI3K)/protein kinase B (PKB/Akt)/glycogen synthase kinase 3 beta (GSK-3β) signaling pathway related proteins were measured. *In vitro*, apoptosis, mitochondrial superoxide, mitochondrial membrane potential, mitochondrial morphology, levels of mitochondrial fission/fusion associated proteins, mitochondrial respiratory function, and AMP-activated protein kinase (AMPK) activity were assessed. To further elucidate the regulating effects of SMI on AMPK and PI3K/Akt/GSK-3β signaling pathway, compound C and LY294002 were utilized. *In vivo*, SMI decreased mortality rate, levels of creatine kinase, and creatine kinase-MB. SMI significantly prevented DOX-induced cardiac dysfunction and apoptosis, decreased levels of Bax/Bcl-2 and cleaved-Caspase3, increased levels of PI3K, p-Akt, and p-GSK-3β. *In vitro*, SMI rescued DOX-injured H9c2 cardiomyocytes from apoptosis, excessive mitochondrial reactive oxygen species production and descending mitochondrial membrane potential, which were markedly suppressed by LY294002. SMI increased ratio of L-OPA1 to S-OPA1, levels of AMPK phosphorylation, and DRP1 phosphorylation (Ser637) in order to prevent DOX-induced excessive mitochondrial fission and insufficient mitochondrial fusion. In conclusion, SMI prevents DOX-induced cardiotoxicity, inhibits mitochondrial oxidative stress and mitochondrial fragmentation through activation of AMPK and PI3K/Akt/GSK-3β signaling pathway.

## Introduction

Doxorubicin (DOX) is one of the most effective anti-cancer agents. Despite of its efficacy on lymphoma and leukemia treatment, its clinical use is limited by severe cardiotoxicity. During the first year of therapy, cardiotoxicity of DOX occurs and mainly presents with cardiomyocyte death (Vejpongsa and Yeh, 2014; Cardinale et al., 2015), which is the main cause of non-cancerous morbidity and mortality in cancer patients after chemotherapy (Dillenburg et al., 2013). Dexrazoxane (DRZ), as the only Food and Drug Administration approved drug to alleviate DOX-induced cardiotoxicity, has not been preferred because of its myelosuppression effect (Seifert et al., 1994). It is necessary to find cardioprotective drugs with low toxicity to combine with DOX in cancer treatment.

Mitochondria are the main target organelle of myocardium damaged by DOX. Accumulating evidence indicates that DOX facilitates cardiomyocyte apoptosis and death through damaging mitochondrial structure and function, which is attributed to disturbance of mitochondrial oxidation-reduction homeostasis and mitochondrial dynamic. DOX causes mitochondrial ROS production as well as oxidative stress, and thereby impairs mitochondrial membrane structure, depolarizes mitochondrial membrane potential, which triggers apoptosis (Caso et al., 2017). Recent reports have confirmed that DOX caused excessive mitochondrial fragmentation characterized by upregulation of dynamin-related protein-1 (DRP1) phosphorylation and downregulation of optic atrophy 1 (OPA1), which promotes mitochondrial-dependent apoptosis in cardiomyocytes (Catanzaro et al., 2019; Wan et al., 2019). In particular, GSK-3β is a downstream effector of PI3K/Akt signaling pathway and can lead to the mitochondrial permeability transition pore (mPTP) opening (Nishihara et al., 2007). Phosphorylation of GSK-3β prompts cell to resist mPTP opening and subsequently apoptosis. Moreover, AMP-activated protein kinase (AMPK) plays a role in regulating mitochondrial dynamics. It inhibits mitochondrial fission through phosphorylation of DRP1 at Ser637.

Shenmai injection (SMI), containing extracts of *Panax ginseng* C.A.Mey and *Ophiopogon japonicus* (Linn.f.) Ker-Gawl, is widely used medication in the prevention and treatment of cardiovascular disease (CVD) in China (Shi et al., 2015; Xian et al., 2016). And it is often combined with chemotherapeutic drugs to increase their curative effects, reduce their damage to non-cancerous tissue and improve immune function of cancer patients (Liu et al., 2014; Liu et al., 2017; Fang et al., 2018). A clinical evidence has shown that SMI could decrease the incidence of electrocardiogram abnormality and cardiac function abnormality in breast cancer patients with DOX treatment (Liu et al., 2014). Experimental evidences have reported that the cardioprotective efficacy of SMI against DOX is associated with scavenging free radical and relieving calcium overload (Wang and Ma, 2001; Chen L. et al., 2003; Liu et al., 2009). SMI alleviated acute cardiotoxicity induced by DOX *via* regulation of inflammatory mediators (Zhang et al., 2019). Shengmai injection, composed of schisandra chinensis and other two components same as SMI, has been reported to be reflective of the energy disruption and cardiac dysfunction induced by DOX (Chen et al., 2015). Moreover, Ophiopogoni D, one of the main active constituents, could rescue autophagic cell death through attenuating mitochondrial damage in DOX-treated cardiomyocytes (Zhang et al., 2015). Despite of these researches, it was not determined whether SMI could regulate mitochondrial homeostasis in DOX-injured myocardium. Therefore, the purpose of this research was to investigate the underlying protective mechanisms of SMI on DOX-induced myocardial injury.

## Materials and Methods

### Reagents and Chemicals

SMI was purchased from CTQ Pharmaceutical Group Co. Ltd. (Hangzhou, China), the same batch as previous study (Yu et al., 2019). Cell culture supplies were purchased from Gibco (Grand Island, NY, USA). Anti-PI3K, anti-Akt, anti-Phospho-Akt (Ser473), anti-GSK-3β, anti-Phospho-GSK-3β (Ser9), anti-GAPDH, anti-AMPKα, anti-Phospho-AMPKα (Thr172), anti-Phospho-DRP1 (Ser616), anti-Phospho-DRP1 (Ser637), anti-Bax, anti-Bcl-2, anti-Caspase3, and anti-cleaved-Caspase3 were purchased from Cell Signaling Technology (Danvers, MA, USA). Anti-OPA1, anti-MFN2, and anti-FIS1 were purchased from Abcam (Cambridge, MA, UK). Anti-DRP1 and anti-MFN1 were purchased from Santa Cruz Biotechnology (Dallas, Texas, USA). MitoSOX Red, MitoTracker Green, and MitoTracker Deep Red were purchased from Invitrogen (Eugene, USA).

### Animals and Treatment

Adult male (22 ± 1 g) and female (18 ± 1 g) C57BL/6 mice, 6 weeks of age, were purchased from Beijing Vital River Laboratory Animal Technology Co., Ltd. Mice were routinely kept at the animal room of the Tianjin University of Traditional Chinese Medicine. All interventions and animal care procedures were performed in accordance with the Guidance Suggestions for the Care and Use of Laboratory Animals issued by the Ministry of Science and Technology of China. The protocols were approved by the Laboratory Animal Ethics Committee of Tianjin University of Traditional Chinese Medicine (Tianjin, China; Permit NO. TCM-LAEC2018028).

**Figure 1:**
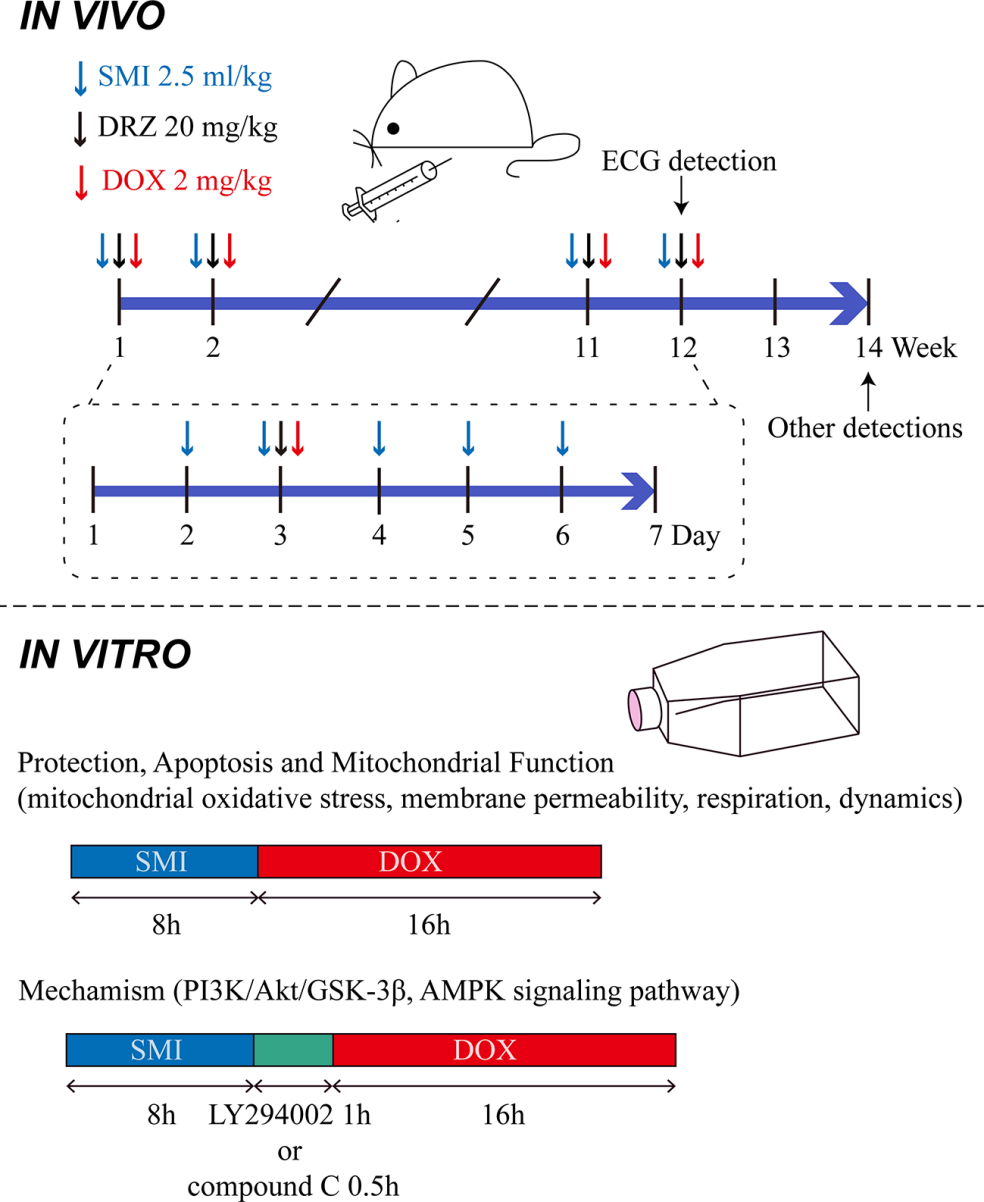
Administration manner *in vivo* and *in vitro*.

Mice were randomly divided into the following four groups, based on their body weight: control group (Control), DOX injury group (DOX), SMI treatment group (DOX + SMI), DRZ treatment (20 mg/kg) group (DOX + DRZ). Mice in SMI treatment group were administrated with SMI (2.5 ml/kg body weight, i.p.) from day 2 to 6 each week. Mice in DRZ treatment group were administrated with DRZ (2.5 ml/kg body weight, i.p., 250 mg dissolved in 25 ml sodium lactate solution and 6.25 ml normal saline) on day 3 each week. Mice except Control group were administrated with DOX (2 mg/kg body weight, i.p.) on day 3 each week 30 min after the first administration. Normal saline was given as a control. In accordance with the previous methods (Vandenwijngaert et al., 2017), administrations lasted for 12 weeks. The accumulative dosage of DOX was 24 mg/kg body weight. Dose of 2.5 ml/kg of SMI equated to 1 × the human equivalent dose. The above administration manner was descripted in **Figure 1**.

### Echocardiography Evaluation of Left Ventricular Function

Left ventricular function was evaluated by transthoracic echocardiography and a Vevo 2100 ultra-high resolution small animal ultrasound imaging system (Fujifilm VisualSonics, Toronto, ON, Canada) at the end of the experiment. Anesthesia was performed by inhalation of isoflurane (1% oxygen plus 5% isoflurane for induction and 1% oxygen plus 2% isoflurane for maintenance). Left ventricular end-systolic and end-diastolic diameters, including left ventricular volume systole (LV Vol,s), left ventricular ejection fraction (EF %) and fractional shortening (FS %) were measured.

### Survival Rate of Mice

Mortality of mice was recorded every day. Rate of survival was traced at the end of the experiment, which was expressed as (total number of experimental animals-number of dead animals)/total number of experimental animals*100%.

### Measurement of Markers of Myocardial Injury

At the end of the experiment, mice were anaesthetized with tribromoethanol solution (2%, 0.1 mL/10 g body weight, i.p.). When mice were unconscious, blood was sampled from orbital veil to examine the level of creatine kinase (CK) and creatine kinase-MB (CK-MB) by an automatic biochemical analyzer (Thermo Fisher Scientific).

### Histopathological Examination

After anesthetized mice were euthanized by cervical dislocation, heart was collected immediately. Left ventricle was routinely fixed and embedded in paraffin according to a standard protocol. The tissue was sectioned and then stained with hematoxylin-eosin (H&E) for histopathological analysis.

### Terminal Deoxynucleotidyl Transferase-Mediated dUTP Nick End Labeling (TUNEL) Staining

TUNEL staining assay was performed following instructions of *in situ* apoptosis detection kit (Roche, Mannheim, Germany). A fluorescence microscope (Zeiss, Waltham, MA) was used for obtaining images from three random areas of three sections per mouse. The apoptosis index was expressed as the percentage of the number of TUNEL-positively stained nuclei to the number of 4,6-diamidino-2-phenylindole (DAPI)-stained nuclei.

### Wheat Germ Agglutinin (WGA) Staining

Mean cardiomyocyte cross-sectional area was detected in deparaffinized sections stained with fluorescein isothiocyanate (FITC)-labeled WGA (1:100, Invitrogen, Grand Island, NY). Nuclei were counterstained with DAPI. Images were acquired using the fluorescence microscope and analyzed by ImageJ 1.47v software (Wayne Rasband, Maryland, USA).

### Cell Culture and Treatment

Rat embryonic ventricular myocardial cell line H9c2 was purchased from the American Type Cell Culture (ATCC, Manassas, VA). Cells were cultured as previously described (Li et al., 2017). Briefly, H9c2 cardiomyocytes were cultured in DMEM medium with the addition of 10% fetal bovine serum and 1% penicillin/streptomycin at 37°C in a humidified incubator containing 5% CO_2_. SMI was diluted with DMEM medium. DOX was dissolved in DMSO at a stock concentration of 25 mM. To verify cardioprotective effect of SMI, cells were pretreated with SMI (0.5%, 0.125%, 0.032%, 0.004%) for 8 h. Afterward, DOX (1 μM) was added for another 16 h. For detection of apoptosis and mitochondrial function, cells were pretreated with SMI (0.5%, 0.125%) for 8 h prior to DOX treatment (1 μM, 16 h). For inhibitor experiments, cells were pretreated with PI3K inhibitor (LY294002, 10 μM, 1 h) and AMPK inhibitor (compound C, 10 μM, 0.5 h) respectively, before DOX treatment (**Figure 1**) (Park et al., 2014; Li et al., 2017).

### Cell Viability Assay

Cell viability was determined using a CCK8 assay. Cells were incubated with CCK8 solution (Dojindo, Kumamoto, Japan) at 37°C for 3 h at the end of the treatment. Absorbance was measured at 450 nm using a microplate reader (Tecan, Sunrise, Austria).

### Hoechst 33342 Staining

Cells were incubated with 10 μg/ml of Hoechst 33342 (Sigma, St. Louis, MO) dye for 15 min at 37°C. Images were obtained using a fluorescence microscope (Zeiss, Waltham, MA).

### Annexin-V/PI Staining

Cells were harvested and stained with FITC-conjugated Annexin V and PI using FITC Annexin V Apoptosis Detection Kit (BD, CA, USA). The rate of apoptosis was analyzed using a flow cytometer (BD, NJ, USA).

### Measurement of Mitochondrial Membrane Potential (ΔΨm)

Cells were incubated with JC-1 (Beyotime Biotechnology, Shanghai, China) according to the instruction. Images were obtained using a fluorescence microscope (Zeiss, Waltham, MA). Fluorescence intensity was detected by a fluorescence microplate reader (Molecular Devices, San Jose, CA) (emission at 515 and 585 nm; excitation at 529 and 590 nm).

### Measurement of Mitochondrial Superoxide

Cells were incubated in Hank’s buffer with MitoSOX Red (5 μM) at 37°C for 10 min and then with the mitochondrion-selective probe MitoTracker Green (200 nm) for 15 min. Images were taken with a fluorescence microscope (Zeiss, Waltham, MA). Fluorescence intensity was calculated using ImageJ 1.47v software (Wayne Rasband, Maryland, USA).

### Analysis of Mitochondrial Respiration

Mitochondrial oxygen consumption rate (OCR) was determined using Seahorse XFe24 Analyzer (Seahorse Biosciences, North Billerica, MA). Cells were seeded at a density of 8,000/well into 24 wells of Seahorse XF24 cell culture microplates. After treatment, medium was changed 1 h before the start of the extracellular flux assay to assay medium. Cells were sequentially treated with oligomycin (1 μM), carbonyl cyanide p-tri-fluoromethocyphenylhydrazone (FCCP, 2 μM), rotenone (0.5 μM), and antimycin A (0.5 μM). OCR was calculated using Seahorse software.

### Mitochondrial Fission Analysis

In H9c2, mitochondria were labeled by MitoTracker Deep Red. Images were obtained with the confocal microscope (Zeiss LSM 700, Waltham, MA). Image J 14.1o software was used for calculating the aspect ratio (AR) and form factor (FF) to evaluate mitochondrial morphology according to the previous study (Bai et al., 2013). In brief, the fluorescent images were converted to binary images. Mitochondrial particles were determined for length, width, perimeter, and area. AR was calculated as the ratio of the major to minor axes of the ellipse to assess the length of the mitochondria. FF was used for determining the degree of mitochondrial branching, and calculated as the following equation: FF = Perimeter^2^/4π × area. Ten cells were imaged and analyzed from three confocal dishes.

### Western Blotting Assay

The whole protein of the left ventricle or H9c2 cells was extracted from three samples in each group. Equal amounts of protein were separated *via* sodium dodecyl sulfate-polyacrylamide gel electrophoresis (SDS-PAGE), transferred to polyvinylidene difluoride membranes (Millipore, MA, USA), and probed with specific antibodies. The bands were assessed using an electrochemiluminescence (ECL) system.

### Statistical Analysis

Data were expressed as means ± standard deviation (SD). The differences among groups were analyzed with one-way analysis of variance (ANOVA) followed by LSD Method and Dunnett’s C test. The differences of survival rates among groups were analyzed with Logrank Test. *P* < 0.05 was considered statistically significant.

## Results

### SMI Improves DOX-Induced Cardiac Injury in Mice

**Figure 2:**
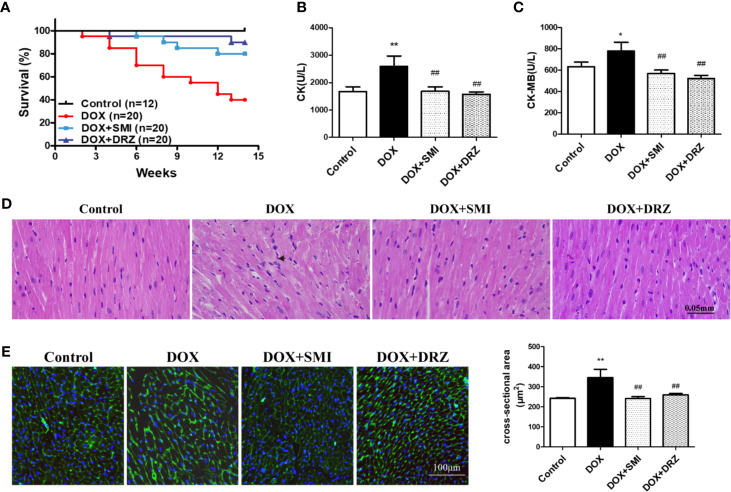
Effect of SMI on DOX-induced cardiac injury in mice. **(A)** Survival curves (n = 12 for the control group, n = 20 for the other groups). **(B, C)** The levels of CK and CK-MB in serum (n = 12 for the control group, n = 8 for the DOX injury group, n = 16 for the SMI treatment group, n = 18 for the DRZ treatment group). **(D)** HE staining of myocardial sections. Immune cells are marked with short arrows. **(E)** WGA staining of myocardial sections (n = 3). Ventricular cardiomyocyte cross-sectional area measurements (right). The value represents the mean ± SD. ^*^P < 0.05, ^**^P < 0.01 vs. control group, ^##^*P* < 0.01 vs. DOX injury group.

C56BL/6 mice were administrated with DOX for 12 w with 24 mg/kg body weight accumulative dosage to mimic DOX-induced chronic myocardial damage. DRZ was utilized as a positive control. As shown in **Figure 2A**, no mice in the control group died. At 14 w, the survival rate of mice in the DOX injury group, DRZ and SMI treatment group was 40%, 90%, and 80%, respectively. Over the course of 14 w, survival rate of mice treated with DOX was significantly lower than that of normal mice (*P* < 0.01). However, this decreased survival rate of mice by DOX was significantly inhibited by SMI and DRZ treatment (*P* < 0.01). Elevated levels of CK and CK-MB in serum indicate the loss of cardiomyocyte structural integrity. As depicted in **Figures 2B, C**, both CK and CK-MB levels were markedly increased by the DOX (*P <*0.01, *P <*0.05). However, there were significantly decreased CK and CK-MB levels in serum of SMI and DRZ treatment group compared to DOX injury group (*P* < 0.01).

To visually detect myocardial injury, H&E and WGA staining were performed for the histopathological examination. As shown in **Figure 2D**, DOX administration caused disorganization of myofibrillar arrays and infiltration of immune cells, which were restored by SMI. Consistently, myocardium in the DOX group presented increased intercellular space and membrane disappearance (**Figure 2E**). However, shape of myocardium in SMI treatment group was close to that in control group.

### SMI Improves DOX-Induced Cardiac Dysfunction in Mice

**Figure 3:**
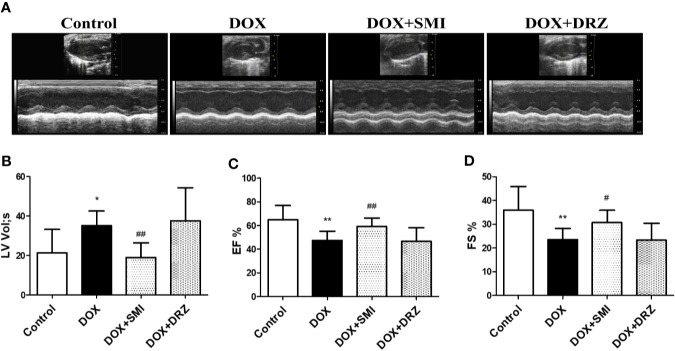
SMI improves DOX-induced cardiac dysfunction in mice. **(A)** Representative M-mode echocardiograms. **(B–D)** Left ventricular volume systole (LV Vol,s), left ventricular ejection fraction (EF %) and fractional shortening (FS %) were evaluated. The values represent the mean ± SD, with n = 12 for the control group, n = 8 for the DOX injury group, n = 16 for the SMI 2.5 ml/kg treatment group, n = 18 for the DRZ treatment group. ^*^P < 0.05, ^**^P < 0.01 vs. control group, ^#^*P* < 0.05, ^##^*P* < 0.01 vs. DOX injury group.

As depicted in **Figure 3**, echocardiographic analysis demonstrated that EF and FS were much lower in DOX-injured mice than in normal mice (*P* < 0.01). However, the decreased EF and FS was markedly rescued by SMI treatment (*P* < 0.01, *P* < 0.05). Consistently, the increased LV by DOX was largely attenuated by SMI treatment (*P <* 0.01). EF, FS, and LV levels were comparable between DOX-injured mice and DRZ-treated mice, which was inconsistent with results of CK and CK-MB detection. ECG was performed at 12th week after DOX injury, while serum enzymatic indicators were detected after 2 weeks of recovery. Therefore, the inconsistency indicated that the cardiac protective effect of DRZ was more obvious after convalescence.

### SMI Reverses the Changes in DOX-Induced Apoptosis in Mice and H9c2 Cell

**Figure 4:**
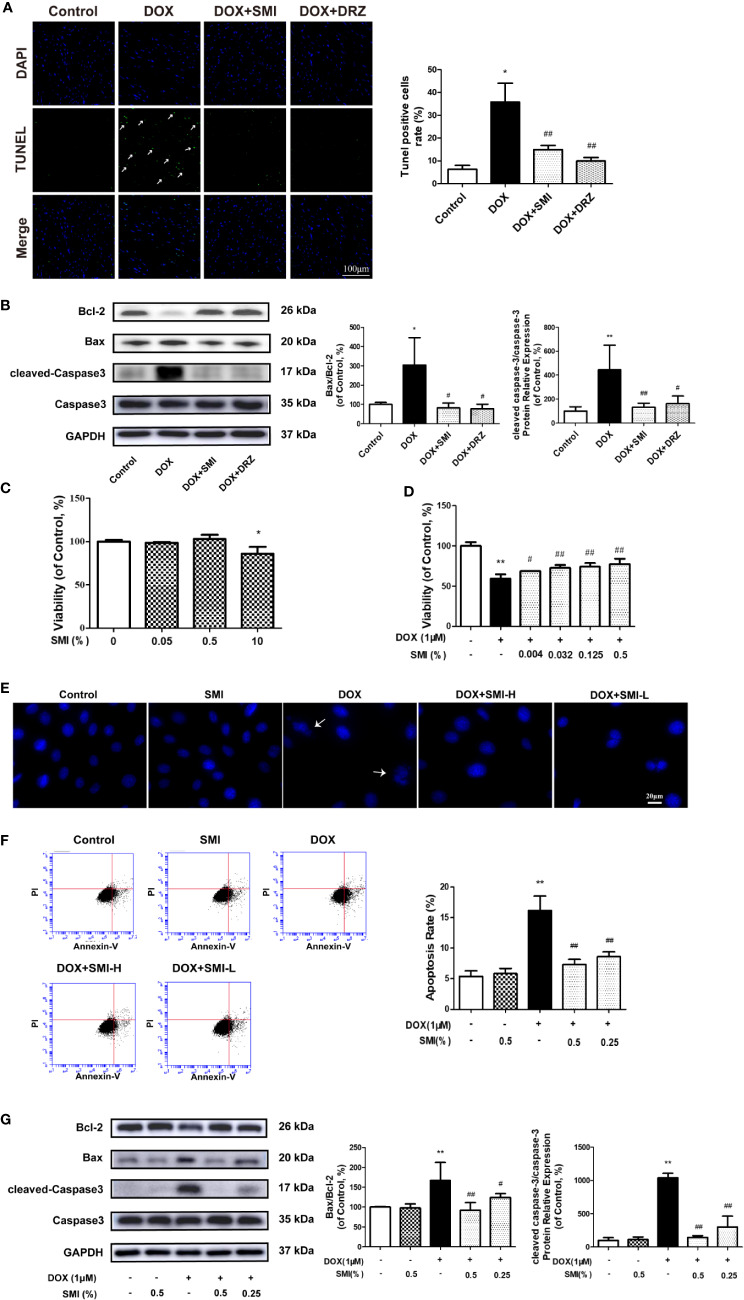
SMI reverses the changes in DOX-induced apoptosis in mice by TUNEL assay, and in H9c2 cell by CCK8 assay, Hoechst 33342 and Annexin V/PI staining. **(A)** TUNEL staining of myocardial sections. Representative images are shown. Nuclei of apoptosis cells are marked with white arrows. **(B)** Western blot analysis of Bcl-2, Bax, cleaved-Caspase-3 in cardiac homogenates prepared from mice. **(C)** CCK8 assay for detecting the viable effect of SMI on normal cells. Cells were treated with SMI (10%, 0.5%, 0.05%) for 8 h. **(D)** CCK8 assay for detecting the protective effect of SMI on DOX-injured cells. Cells were pretreated with SMI (0.5%, 0.125%, 0.032%, 0.004%) for 8 h prior to DOX treatment (1 μM, 16 h). **(E–G)** Cells were pretreated with SMI-H (0.5%) and SMI-L (0.25%) for 8 h, and then stimulated with DOX (1 μM) for 16 h. **(E)** Representative images of Hoechst33342 positive cells. Damaged nuclei are marked with white arrows. **(F)** Annexin V/PI staining for apoptosis detection. **(G)** Western blot analysis of Bcl-2, Bax, cleaved-Caspase-3 in H9c2 cells. The values represent the mean ± SD (n = 3). ^*^P < 0.05, ^**^P < 0.01 vs. control group, ^#^*P* < 0.05, ^##^*P* < 0.01 vs. DOX injury group.

The anti-apoptosis effect of SMI in DOX-treated mice was measured by TUNEL. Consistent with earlier studies (Ma et al., 2017), DOX caused a significant increase in TUNEL-positive cells (*P* < 0.05, **Figure 4A**). The number of TUNEL-positive cells was decreased to 14.92 ± 1.85% and 9.96 ± 1.52% (*P* < 0.01) when mice were treated with SMI or DRZ. Level of cleaved-Caspase3 and ratio of Bax to Bcl-2 were markedly higher in DOX-injured heart homogenates than in the counterparts (*P* < 0.01, *P* < 0.05, **Figure 4B**), whereas these increased level and ratio were significantly suppressed by SMI treatment (*P* < 0.01, *P* < 0.05).

In H9c2 cardiomyocytes, we used CCK8, Hoechst 33342, Annexin V/PI, and immunoblotting of apoptosis-related factors to determine the protective effects of SMI on DOX-induced cardiomyocyte damage and apoptosis. Compared with untreated cells, there was a significantly reduced cell viability when cells were incubated with SMI (10%) (*P* < 0.05). However, no significant difference appeared when cells were incubated with the lower concentrations of SMI (0.5%, 0.05%) (**Figure 4C**), which indicated that SMI (0.5%, 0.05%) had no toxicity on H9c2 cells. Therefore, the maximum dose of SMI for subsequent experiments did not exceed 0.5%.

Then, we further examined the protective effects of SMI against cell injury and apoptosis. As depicted in **Figure 4D**, DOX induced a significant decrease in cell viability (*P* < 0.01), which was alleviated by SMI in a dose-dependent manner. Hoechst 33342 staining assay indicated that DOX injury led to apoptosis in H9c2 cells, presenting with condensed nuclei (**Figure 4E**). However, SMI (0.5%, 0.25%) restored the nuclei to their normal morphology. Early-stage apoptosis was evaluated by Annexin V/PI staining. Consistently, the ratio of Annexin V-positive cells to PI negative cells was significantly increased by DOX (*P* < 0.01, **Figure 4F**). There was a significantly reduced ratio in cells of SMI treatment group (*P* < 0.01, **Figure 4F**). As shown in **Figure 4G**, levels of cleaved-Caspase3 and ratio of Bax to Bcl-2 were significantly increased by DOX treatment (*P* < 0.01), which were attenuated by SMI treatment in a dose-dependent manner (*P* < 0.05, *P* < 0.01).

### SMI Suppresses DOX-Induced Mitochondrial Superoxide Formation in H9c2 Cell

**Figure 5:**
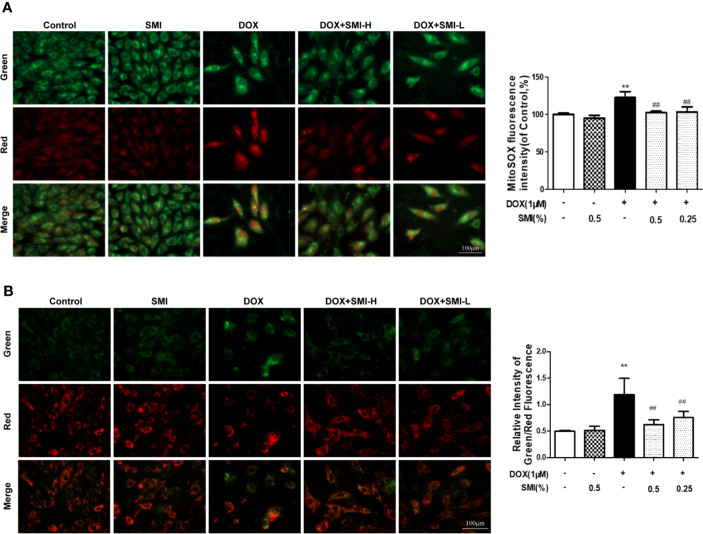
SMI suppresses DOX-induced mitochondrial superoxide formation and mitochondrial integrity injury in H9c2 cell. Cells were pretreated with SMI-H (0.5%) and SMI-L (0.25%) for 8 h, then stimulated with DOX (1 μM) for 16 h. **(A)** Representative images of mitochondria, mitochondrial superoxide, and merges. Quantitation of MitoSOX by fluorescence intensity. **(B)** Representative images of cells with JC-1 staining. Quantitation of mitochondrial membrane potential by ratio of green to red fluorescence intensity. The values represent the mean ± SD (n = 3). ^**^P < 0.01 vs. control group, ^##^*P* < 0.01 vs. DOX injury group.

Cells were dual-labeled with MitoSox Red and MitoTracker Green, which labels superoxide anion in mitochondria and mitochondria regardless of the polarization state. As shown in **Figure 5A**, SMI (0.25%, 0.5%) significantly attenuated the increase in mitochondrial superoxide anion (red) induced by DOX treatment.

### SMI Attenuates DOX-Induced Mitochondrial Integrity Injury in H9c2 Cell

To investigate the mitochondrial membrane potential, we utilized JC-1 probe. As shown in **Figure 5B**, DOX treatment led to a significant increase in the ratio of green to red fluorescence intensity, indicating a significant reduction of mitochondrial membrane potential. As expected, SMI pretreatment markedly suppressed this increased ratio, suggesting that SMI could rescue DOX-induced loss of mitochondrial membrane potential.

### SMI Modulates DOX-Induced Mitochondrial Respiratory Dysfunction in H9c2 Cell

**Figure 6:**
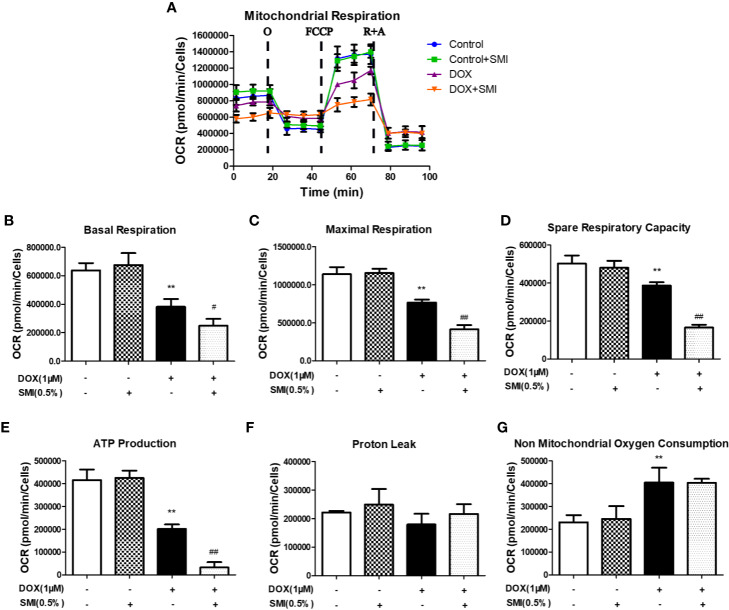
SMI modulates DOX-induced mitochondrial respiratory dysfunction in H9c2 cell. Cells were pretreated with SMI (0.5%) for 8 h, then stimulated with DOX (1 μM) for 16 h. **(A)** OCR in H9c2 cells was monitored using a Seahorse metabolic analyzer, following the addition of oligomycin (O), FCCP and rotenone/antimycin (R + A). **(B–G)** Basal respiration, maximal respiration, spare respiratory capacity, ATP production, proton leak, and non-mitochondrial oxygen consumption were quantified. The values represent the mean ± SD (n = 3). ^**^P < 0.01 vs. control group, ^#^*P* < 0.05, ^##^*P* < 0.01 vs. DOX injury group.

To further investigate the mechanism responsible for the protective effects of SMI, we assessed mitochondrial respiration using a Seahorse Bioscience extracellular flux analyzer. DOX significantly reduced the basal respiration, maximal respiration, spare respiratory capacity, and ATP production to 60%, 67%, 77%, and 48% of those in the control group (*P* < 0.01, **Figures 6A, B, C, D, E**). There was no change of proton leak (**Figures 6F**) and a markedly increased level of non mitochondrial oxygen consumption (**Figures 6G**) in DOX-injured H9c2 cells. To our surprise, these four parameters for the SMI pretreatment were significantly lower than those of the DOX injury group, which indicated that mitochondrial respiration was inhibited by the SMI in DOX-injured H9c2 cells.

### SMI Activates PI3K/Akt Signaling Pathway in DOX-Injured Mice and H9c2 Cell

**Figure 7:**
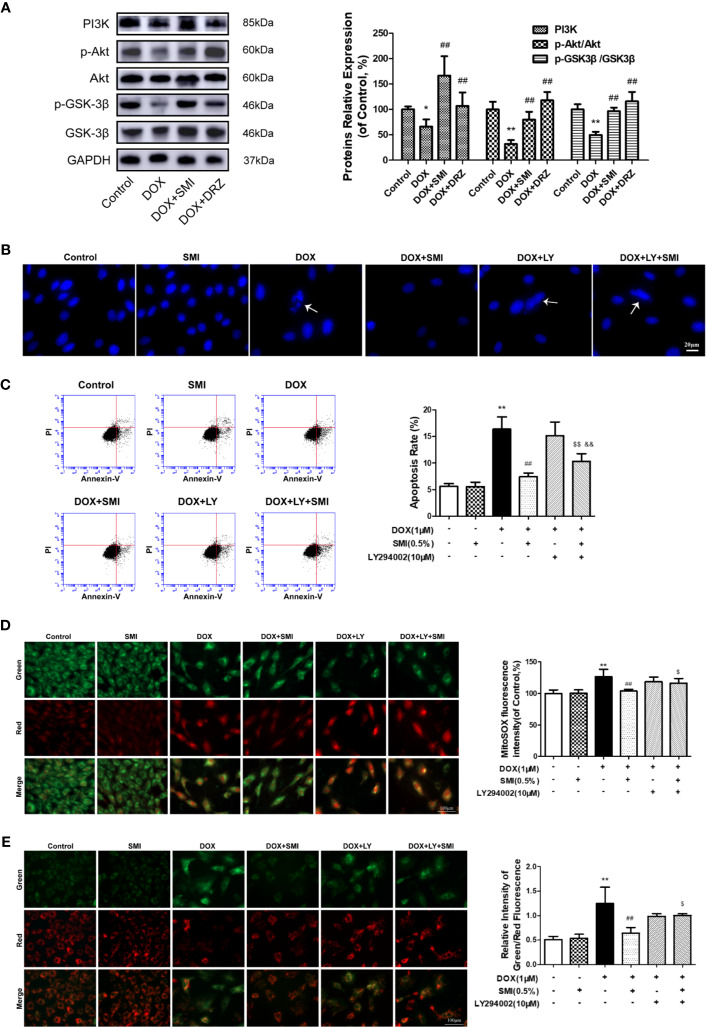
SMI activates PI3K/Akt signaling pathway in DOX-injured mice and H9c2 cell. **(A)** Western blot analysis of PI3K, phosphorylated Akt at Ser473 (p-Akt) and phosphorylated GSK-3β at Ser9 (p-GSK-3β) in cardiac homogenates prepared from mice. **(B–E)** Cells were pretreated with SMI (0.5%) for 8 h, LY294002 (10 μM) for 1 h, then stimulated with DOX (1 μM) for 16 h. **(B)** Hoechst 33342 staining. Damaged nuclei are marked with white arrows. **(C)** Representative images of Annexin V/PI staining and quantitative data of the ratio of Annexin-V positive cells to PI negative cells. **(D)** Representative images of mitochondria, mitochondrial superoxide and merges. Bar diagram showing the MitoSOX fluorescence intensity. **(E)** Representative images of cells with JC-1 staining. Quantitative data of the ratio of green to red fluorescence intensity (right). The values represent the mean ± SD (n = 3). ^*^P < 0.05, ^**^P < 0.01 vs. control group, ^##^*P* < 0.01 vs. DOX injury group, ^$^*P* < 0.05, ^$$^*P* < 0.01 vs. SMI treatment group, ^&&^*P* < 0.01 vs. DOX+LY294002 treatment group.

PI3K/Akt signaling pathway participates in inhibiting of DOX-induced myocardial damage. To investigate whether pretreatment with SMI activated PI3K/Akt signaling pathway in DOX-injured mice myocardium, we assessed levels of PI3K, p-Akt, and p-GSK-3β using Western blot. As depicted in **Figure 7A**, levels of PI3K, p-Akt, and p-GSK-3β were significantly suppressed in DOX-treated mice (*P* < 0.05, *P* < 0.01), whereas these decreased levels were significantly reversed by SMI treatment (*P* < 0.01).

To further verify whether PI3K/Akt signaling pathway contributed to SMI cardioprotective effect, we utilized a PI3K-specific inhibitor LY294002 in H9c2 cells. As shown in **Figures 7B, C**, LY294002 markedly diminished the anti-apoptotic effect of SMI, presenting increased number of condensed nuclei and ratio of Annexin V positive to PI negative cells. In the meanwhile, LY294002 markedly suppressed regulating effects of SMI on fluorescent intensity of MitoSOX and mitochondrial membrane potential (**Figures 7D, E**, *P* < 0.05).

### SMI Alleviates DOX-Induced Mitochondrial Fragmentation in H9c2 Cell

**Figure 8:**
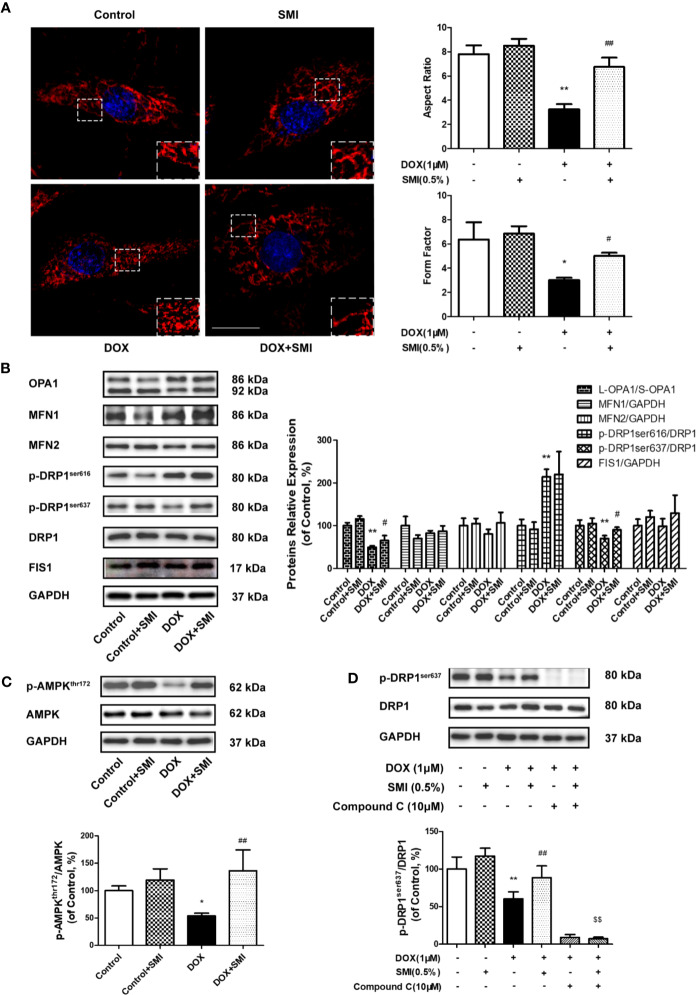
SMI alleviates DOX-induced mitochondrial fragmentation in H9c2 cell. **(A, B)** Cells were pretreated with SMI (0.5%) for 8 h, then stimulated with DOX (1 μM) for 16 h. **(A)** Mitochondria was labeled with MitoTracker Deep Red. Representative confocal microscopy image is shown. Scale bar: 25 μm. Quantification of mitochondrial fission with Aspect Ratio and Form Factor. **(B)** The relative protein expression of L-OPA1/S-OPA1, MFN1, MFN2, phosphorylated DRP1 at Ser616 and Ser 637, DRP1, and FIS1. **(C)** The relative protein expression of phosphorylated AMPK at Thr172 and AMPK. **(D)** Cells were pretreated with SMI (0.5%) for 8 h, compound C (10 μM) for 0.5 h, then stimulated with DOX (1 μM) for 16 h. The relative protein expression of phosphorylated DRP1 at Ser616, DRP1. The values represent the mean ± SD. (n = 3). ^*^P < 0.05, ^**^P < 0.01 vs. control group, ^#^*P* < 0.05, ^##^*P* < 0.01 vs. DOX injury group, ^$$^*P* < 0.01 vs. SMI treatment group.

To determine the impact of SMI on DOX-induced mitochondrial fragmentation, we examined the mitochondrial shape. As shown in **Figure 8A**, normal cells presented elongated and interconnected mitochondria, while much shorter and smaller mitochondria were widespread in DOX-injured cells. However, shape of mitochondria in SMI pretreatment group was close to that in normal group. Then, AR and FF were used for further quantitative evaluation of mitochondria shape. As depicted in **Figure 7A**, AR and FF value were much lower in DOX-injured cells than in normal cells, while the decreased AR and FF value was largely rescued by SMI pretreatment.

Mitochondrial fragmentation is attributed to an imbalance between fission and fusion. Thus, we further examined whether SMI had an effect on the levels of mitochondrial dynamics proteins. As shown in **Figure 8B**, the levels of fusion-related protein, including MFN1 and MFN2, were generally unchanged by DOX, while ratio of long form of OPA1 (L-OPA1) to short OPA1 (S-OPA1) was significantly reduced by DOX (**Figure 7B**, *P* < 0.01). There was a significantly increased ratio of L-OPA1 to S-OPA1 in cells of SMI pretreatment group compared to DOX treatment group (*P* < 0.05). Levels of fission-related protein were shown in **Figure 8B**. DOX led to significantly increased phosphorylation of DRP1 at Ser616 (*P* < 0.01), together with a decreased DRP1 Ser637 phosphorylation (*P* < 0.01). Levels of FIS1 were unchanged in DOX-injured cells. There was no significant change of DRP1 Ser616 in DOX-injured cells after SMI treatment, but significantly increased phosphorylation of DRP1 at Ser637 (*P* < 0.05).

AMPK promotes the phosphorylation of DRP1 at Ser637, which could inhibit DRP1 oligomerization to suppress fission. Compared to that in control group, decreased phosphorylation level of AMPK (Thr172) was detected in DOX-treated cells (*P* < 0.05, **Figure 8C**), while SMI significantly alleviated this decrease (*P* < 0.01). To further confirm SMI activated DRP1 phosphorylation at Ser637 by AMPK activation, we used AMPK inhibitor compound C. As depicted in **Figure 8D**, compound C significantly inhibited SMI’s effect on DPR1 phosphorylation at Ser637 (*P* < 0.01). Taken together, our results demonstrated that SMI suppressed DOX-induced mitochondrial fragmentation, which might be associated with AMPK activation.

## Discussion

DOX-induced cardiac dysfunction is attributable to elevated oxidative stress and mitochondrial dysfunction. SMI is commonly used in the treatment of CVD and is often combined with DOX in clinic to increase its anti-tumor effects and reduce cardiotoxicity. Although experimental evidences have shown that SMI protected myocardium against DOX *via* scavenging free radical and relieving calcium overload (Wang and Ma, 2001; Chen L. et al., 2003; Liu et al., 2009), it is not clear if the protective effect is associated with maintaining mitochondrial homeostasis. The present study confirmed the cardioprotective effect of SMI on DOX-induced myocardial injury both *in vivo* and *in vitro*. Our findings further suggested that its protective mechanism might be attributed to activating AMPK and PI3K/Akt signaling pathway, so that regulated mitochondrial oxidative stress and dynamics, and thereby inhibited apoptosis.

Myocardial injury caused by DOX includes both acute and chronic injuries. Chronic cardiotoxicity of DOX is more common than acute one in clinic (Kalyanaraman, 2020). It manifests progressive myocardial dysfunction after DOX administration and presents development of left ventricular systolic dysfunction (Menna et al., 2008). With the continuous dosage accumulation, the histopathological hallmarks appear, including cytoplasmic vacuolization with the myocytes, myofibrillar disarray and mitochondria swelling (Ferrans et al., 1997). In addition, cardiomyocyte hypertrophy appears to some extent (Mouli et al., 2015; Huang X. et al., 2019). DRZ is a well-recognized cardioprotective agent for the treatment of anthracycline-induced cardiotoxicity. It has been reported to alleviate DOX cardiotoxicity through replacing the iron in the iron-DOX complex (Trajković et al., 2007). In the current study, both SMI and DRZ exhibited strong cardioprotective effects on chronic injured myocardium by DOX, with promotion of survival, improvement of left ventricular function, suppression of pathological injury and alleviation of cardiac hypertrophy. Moreover, since SMI used in the present study was the same batch as our previous study, its quality control could be referred to the previous one (Yu et al., 2019).

Oxidative stress is a vital factor in the progression of DOX damage and mitochondrial dyshomeostasis in cardiac cells. DOX can be transformed to semiquinone, and then reacted with oxygen to create superoxide anions. In the meanwhile, DOX chelates free iron to form an iron-DOX complex, thereby leading to ROS generation (Cappetta et al., 2017). DOX accumulates in the mitochondria and damages the mitochondria electron chain, which finally leading to enhanced production of ROS (Du et al., 2019). Therefore, DOX-induced ROS accumulation is a major reason for cardiotoxicity. Consistent with these findings, our results suggested that DOX dramatically promoted mitochondrial O_2_^−^· production, which was attenuated by SMI pretreatment.

Oxidative stress damages mitochondrial membrane permeability which leads to myocardial dysfunction. In the presence of excessive ROS leakage, mitochondrial inner membrane anion channel can be activated. Simultaneously, it contributes to mPTP opening (Li et al., 2017), which facilitates decrease of mitochondrial membrane potential. These changes trigger apoptosis through the mitochondria-dependent pathway (Huang K. Q. et al., 2019). In our study, both *in vivo* and *in vitro* studies have demonstrated that DOX induces apoptosis, which could be relieved by SMI treatment, due to alleviation of mitochondrial depolarization and decreased mitochondrial membrane potential.

Mitochondria are the main site for ROS production (Townsend et al., 2007). When DOX accumulates, ROS are generated by diminishing the redox cycle at complex I of the electron transport chain (ETC), resulting in the blockage of ATP synthesis (Alexieva et al., 2014; Wen et al., 2019). Consistent with previous studies, our data showed that DOX treatment triggered an elevated ROS production and a declined ATP generation, which might be due to ETC damage. Nevertheless, an interesting finding of our research was that SMI significantly suppressed ATP production in DOX-treated H9c2 cardiomyocytes, although it exhibited protective effect on mitochondrial membrane potential as well as inhibitive effect on oxidative stress. Of note, during the mitochondrial oxidative phosphorylation, with the production of ATP, 1–2% of the O_2_ consumed by mitochondria receives electron leaked by the components of the mitochondrial ETC and gets converted into ROS (Chen Q. et al., 2003; Tremblay and Delbes, 2018). In addition, a previous study has found that Ginseng or ginsenosides may present neuroprotective activity by reducing formation of ROS and ATP through suppressing the ATP synthase activity (Kong et al., 2018). Accordingly, a possible explanation for the suppression of ATP production is that SMI might inhibit mitochondrial oxidative phosphorylation thereby suppressing ROS formation.

Several evidences support a protective role of PI3K/Akt signaling pathway in DOX-induced cardiac dysfunction (Shaw and Cantley, 2006; Hu et al., 2019). The activation of PI3K/Akt signaling pathway could suppress cardiomyocyte apoptosis in DOX-injured cardiomyocyte. GSK-3β phosphorylation prompts cell resist mPTP opening and subsequently apoptosis (Nishihara et al., 2007). The present study found that SMI increased PI3K protein expression and promoted Akt and GSK-3β phosphorylation in DOX-injured cardiomyocyte. Once a PI3K-specific inhibitor added, this anti-apoptosis effect of SMI was reversed. Moreover, the PI3K-specific inhibitor markedly suppressed the SMI-medicated mitochondrial O_2_^−•^ and mitochondrial membrane potential, suggesting that SMI regulated the mPTP opening through activating PI3K/Akt/GSK-3β.

Mitochondrial fusion and fission processes preserve the functional integrity of mitochondria. Both inhibition of mitochondrial fusion protein and promotion of mitochondrial fission protein result in mitochondrial fragmentation, which facilitates mitochondrial dependent apoptosis (Frank et al., 2001; Qi et al., 2018). Mitochondrial fission mainly triggered by DRP1 recruitment from the cytosol to the OMM, which regulated by DRP1 phosphorylation at both Ser616 and Ser637. DRP1 Ser616 phosphorylation induces DRP1 translocation to mitochondria with the help of FIS1 (Toyoma et al., 2016; Qiu et al., 2019). We found that DOX induced excessive mitochondrial fragmentation and increased DRP1 Ser616 phosphorylation, but no changes of FIS1 level. To our surprise, although SMI inhibited excessive mitochondria fragmentation, it had no obvious effect on the increased DRP1 phosphorylation at Ser616. In particular, AMPK promotes DRP1 phosphorylation at the other site, Ser637. Phosphorylated DRP1 at Ser637 inhibits DRP1 oligomerization and suppresses mitochondrial fission (Hall et al., 2014). Accordingly, we further investigated phosphorylation level of DRP1 at Ser637 and regulating effect of AMPK. Our data revealed that DOX markedly reduced DRP1 Ser637 phosphorylation and AMPK phosphorylation, which was largely restored by SMI treatment. This effect of SMI disappeared when AMPK inhibitor was added to cells. Therefore, it is probably that SMI suppressed excessive fragmentation *via* activating AMPK and promoting DRP1 Ser637 phosphorylation. Notably, despite AMPK could be activated by low ADP : ATP. (Davies et al., 1995; Sanders et al., 2007), our data showed that DOX decreased both ATP production and AMPK phosphorylation, which in accordance with previous studies (Gratia et al., 2012; Wang et al., 2012).

Inhibition of fusion leads to mitochondrial fragmentation. MFN1 and MFN2 modulate mitochondrial fusion for the OMM, while OPA1 for the IMM. OPA1 has two forms: L-OPA1 and S-OPA1. The L-OPA1 (92kDa) regulates IMM fusion; while S-OPA1 (86kDa), less active forms of OPA, occurs to inhibit fusion (Duvezin-Caubet et al., 2006; Ehses et al., 2009). The loss of mitochondrial membrane potential reduced OPA1 activity, thereby disrupting in mitochondrial fusion (Hall et al., 2014). Our data demonstrated no DOX-induced changes in MFN1 and MFN2 protein expression, but a decrease in ratio of L-OPA1 to S-OPA1. Taken together, SMI alleviated the inhibition of mitochondrial fusion caused by DOX probably *via* increasing OPA1 activity.

## Conclusions

**Figure 9:**
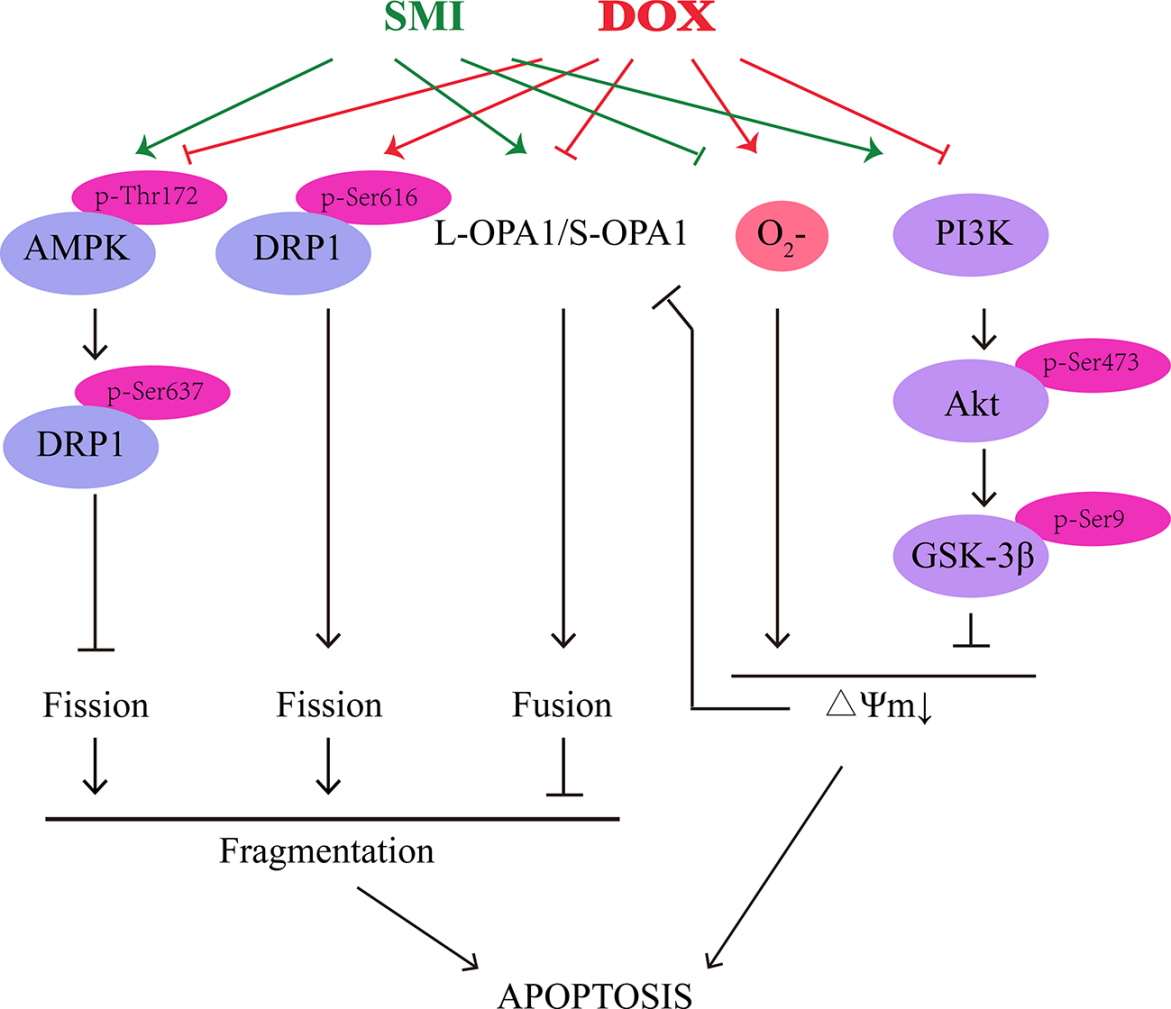
Schematic illustration delineating the mechanism of SMI on DOX-induced cardiotoxicity.

Both in *in vivo* and *in vitro* model of DOX-induced cardiomyopathy and cardiomyocyte apoptosis, we demonstrated that SMI could alleviate DOX-induced cardiotoxicity, improve cardiac function, and maintain mitochondrial homeostasis through activation of AMPK and PI3K/Akt/GSK-3β signaling pathway (**Figure 9**). Further work will be necessary to define how SMI ingredients coordinately regulate signaling pathway to prevent DOX-induced cardiotoxicity.

## Data Availability Statement

All datasets presented in this study are included in the article/**Supplementary Material**.

## Ethics Statement

The animal study was reviewed and approved by the Laboratory Animal Ethics Committee of Tianjin University of Traditional Chinese Medicine (Tianjin, China; Permit NO. TCM-LAEC2018028).

## Author Contributions

LL wrote the original draft. JL, DY, LN, and YY performed the research. JL, XZhe, and XZha analyzed the data. YL, QW, LL, and LH designed the experiment and revised the manuscript.

## Funding

The study was supported by funds from the National Natural Science Foundation of China (81774017, 81830112), the Scientific Research Project of Tianjin Education Commission (2017KJ140 and 2017KJ142), the Natural Science Foundation of Tianjin City (19JCYBJC28200), Tianjin Municipal Health Commission - Research Projects in Key Areas of Traditional Chinese Medicine (2020010), and the Training Program Foundation for Innovative Research Team of Higher Education in Tianjin during the 13th Five-Year Plan Period (No. TD13-5050).

## Conflict of Interest

The authors declare that the research was conducted in the absence of any commercial or financial relationships that could be construed as a potential conflict of interest.
